# Sustainable and efficient monitoring of tryptophan and tyrosine serum levels: a green HPTLC method as a biomarker for type 2 diabetes

**DOI:** 10.1186/s13065-024-01318-9

**Published:** 2024-11-05

**Authors:** Rania M. Kamel, Fatma A. M. Abdel-aal, Fardous A. Mohamed, Abdel-Maaboud I. Mohamed

**Affiliations:** Pharmaceutical Analytical Chemistry Department, Faculty of Pharmacy, Assiut University, Assiut, 71526 Egypt

**Keywords:** Biomarkers, HPTLC, Serum samples, Tryptophan, Type 2 diabetes, Tyrosine

## Abstract

In recent years, there has been considerable interest in using amino acids like tryptophan (Trp) and tyrosine (Tyr) as biomarkers for various diseases, including type 2 diabetes mellitus (T2D). In diseases like T2D, the metabolism of Trp and Tyr is altered. The activity of enzymes involved in Trp metabolism increases, leading to a decrease in its serum level. On the other hand, the serum level of Tyr increases due to the suppressed activity of its metabolizing enzymes. These observations suggest that Trp and Tyr metabolism may play a crucial role in the pathophysiology of type 2 diabetes. Our study highlights the potential utility of Trp and Tyr as biomarkers for the early detection, prognosis, and monitoring of this metabolic disorder. Given these observations, we aimed to develop a high-performance thin-layer chromatographic (HPTLC) method that is sensitive, selective, rapid, and environmentally friendly for estimating the concentrations of Trp and Tyr in biological fluids, particularly serum samples. To evaluate the method, we performed analysis using serum samples from controlled and streptozotocin-induced diabetic rats. Our main objective was to develop a method that is sensitive and selective for precisely determining Trp and Tyr serum levels, which could serve as potential biomarkers for T2D. Fluorescence and absorption modes were employed for densitometry scanning. We assessed the precision and high separation efficiency of the chromatographic system by calculating parameters such as separation and resolution factors, number of theoretical plates, and height equivalent to theoretical plates. To evaluate the environmental impact of our proposed method, we employed the AGREE (Analytical GREEnness metric) and GAPI (Green Analytical Procedure Index) greenness assessment tools. The results confirmed that our method is environmentally friendly and exhibits superior eco-friendliness and greenness compared to other reported methods.

**Graphical Abstract:**

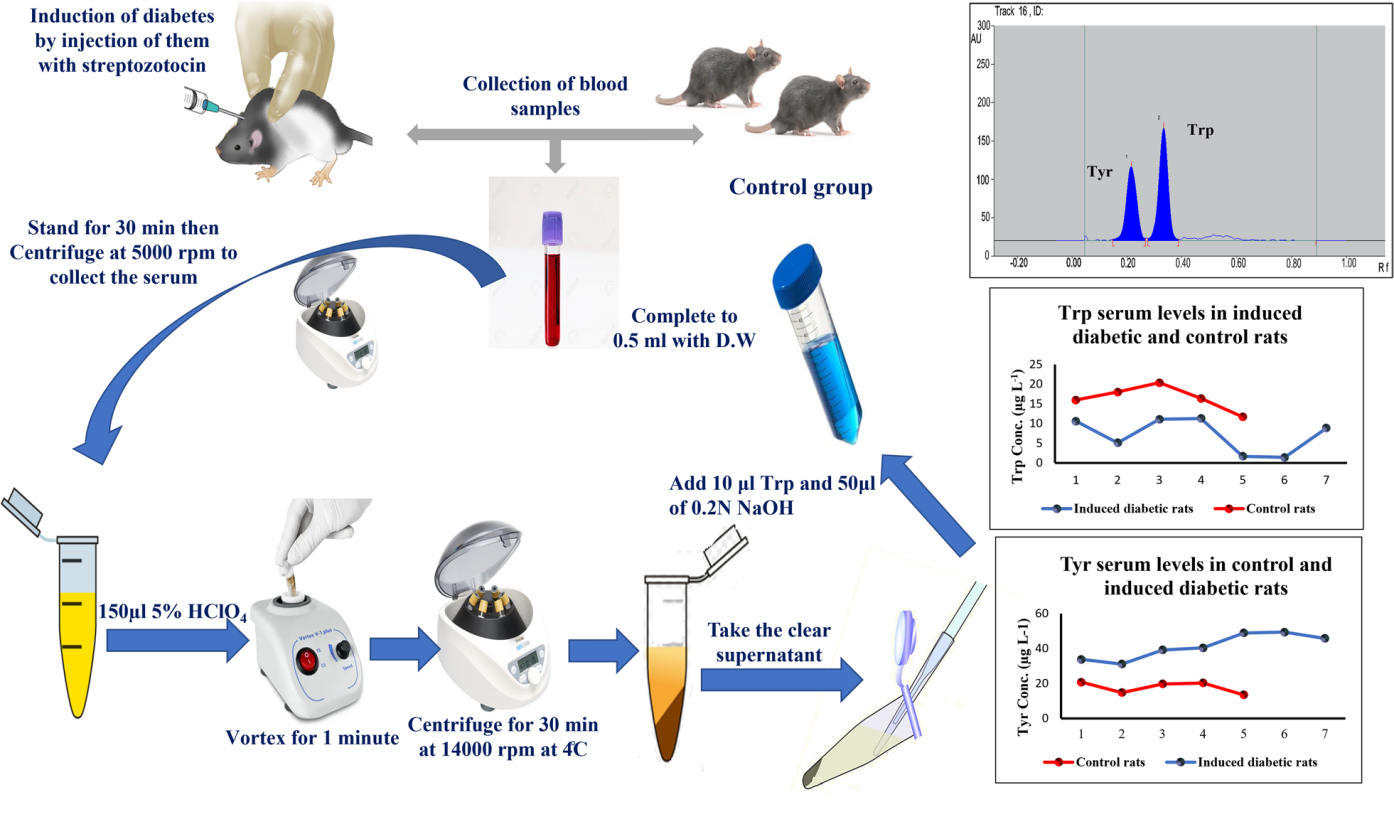

## Introduction

Elevated levels of blood glucose are a hallmark of diabetes, a chronic metabolic disease that can result in severe damage to the eyes, heart, blood vessels, kidneys, and nerves years. Diabetes can be classified into two types: type 1 and type 2. T2D is the most common and usually occurs in adults. It occurs due to the resistance of the body to insulin, or because the body does not produce enough insulin. The prevalence of type 2 diabetes has risen dramatically in countries of all income levels in the last three decades [1]. Nowadays, T2D is not considered a disorder of glucose homeostasis, but also it is related to a number of metabolic dysfunctions including amino acid metabolism [2].

There are several methods for the diagnosis of T2D such as measuring of blood glucose blood levels (fasting plasma glucose test and, oral glucose tolerance test, random plasma glucose test), Glycated Hemoglobin (Hb) A1C and C- peptide test [3, 4]. However, those diagnostic methods have some limitations. Results could be influenced by factors like stress, other diseases and physical activity. Oral glucose tolerance test could be time consuming, less convenient and can lead to miss diagnosis due to alterations in glucose metabolism. While glycated hemoglobin test and C- peptide test could be affected with the diseases such as anaemia and impairments in kidney functions, respectively.

Tryptophan and tyrosine are two types of aromatic amino acids. Tryptophan (Trp) is an essential amino acid, as it cannot be synthesized in the body but should be obtained through diet. On the other hand, tyrosine (Tyr) is considered a semi-essential amino acid as it may be obtained from the diet or synthesized in the body through the hydroxylation of phenylalanine (an essential amino acid).

Trp is metabolized through different pathways but the main pathway is the oxidative (kynurenine) pathway which accounts for around 95% of dietary Trp degradation [5].

Diabetes affects the metabolism of Trp by increasing its degradation by the kynurenine pathway [6]. The activity of indoleamine-2,3-dioxygenase (IDO) (the rate-limiting enzyme for the extrahepatic Trp degradation) increases in T2D cases [7–9]. As diabetic patients are considered to be constantly under stress, the stress conditions also increase the degradation of Trp, mainly through the kynurenine pathway [10].

Tyrosine is metabolized through a pathway involving five enzymes, leading finally to acetoacetate and fumarate [11]. The serum level of Tyr is mainly regulated tyrosine transaminase enzyme [11].

Previous studies showed that Try metabolism is impaired in T2D as it is associated with insulin resistance [12]. A reduction in the expression of the tyrosine transaminase enzyme might lead to an increase in serum tyrosine concentrations. The increase in its concentration might result in inhibition of the insulin signaling pathway and consequently increase insulin resistance [12]. Another mechanism suggested that the increase in tyrosine levels may result in a reduction in tyrosine hydroxylase enzyme activity (the rate-limiting enzyme of catecholamine synthesis), and this may result in a reduction of L-DOPA and dopamine levels and a low anti-incretin effect [13]. Recent studies suggest that dopaminergic agents affect the central nervous system (e.g., Appetit control) and the endocrine system (pituitary and pancreas). Therefore, they are suggested to modulate glucose and energy homeostasis [13, 14].

Biomarkers are characterized as measurable features that indicate normal biological processes, pathogenic processes, or responses to an exposure or intervention.” [15]. Also, the biomarker should possess some criteria such as being safe and easy to measure, cost-saving to follow up on, and modifiable with treatment. Therefore, Trp and Tyr could serve as efficient potential biomarkers to estimate the T2D state.

The use of the HPTLC technique in biomarker assessment was first reported by us. This technique has many advantages, including low operating costs, minimum sample clean-up and high sample throughput. Additionally, several samples can be run up at the same time using small quantities of mobile-phase solvents unlike HPLC, lowering the analysis time and cost.

Therefore, in our present work, we aimed to develop a simple, rapid, sensitive, selective and cost effective validated HPTLC-densitometric method for monitoring Trp and Tyr levels in the serum of control and streptozotocin-induced diabetic rats as a non-invasive method for the detection and screening of diabetes mellitus (DM). Our developed method could be used to evaluate the disease state, and this may be useful in drug discovery for the development of drugs that target the metabolizing enzymes for Trp or Tyr as a treatment for T2D to retain their normal levels and improve the disease state. Also, monitoring of Trp and Tyr levels could be used to predict the near future lifestyle-related diseases including T2D.

## Experimental

### Instrumentation

CAMAG TLC scanner 4 (CAMAG, Muttens, Switzerland) is equipped with CAMAG Linomat 5 for automatic sample application. 100-µL syringe (Hamilton, Bonaduz, Switzerland) for sample application was used under gentle nitrogen steam for the immediate evaporation of solvents. The scanner was operated with WinCATS version 1.4.10 software, which was adjusted in reflectance mode by absorbance or fluorescence using deuterium-tungsten or mercury lamb according to the need. The scanning speed was selected to be 20 mm/s with a data resolution of 100 mm/step. The TLC tank used in the experiment was a standard type with dimensions of 27.0 cm in width, 26.5 cm in height, and 7.0 cm in diameter, sourced from Sigma-Aldrich Co. in St. Louis, MO, USA. HPTLC aluminum plates coated with silica gel 60 (20 × 7 cm) (Merck, Darmstadt, Germany). The ultrasonic cleaner utilized in the experiment was from Cole-Parmer in Chicago, USA. The UV lamp used emitted wavelengths of 254 and 365 nm and was sourced from Vilber Lourmat in Marne-la-Vallée, Cedex, France, operating at 220 V and 50 Hz. Laboratory centrifuge (Hettich,Germany) and sartorius handy balance H51 (Hanover, Germany) were utilized.

### Chemicals and reagents

Trp and Tyr standards were purchased from Sigma Aldrich (Steinheim, Germany) (> 98%). Methanol, ethyl acetate, and acetonitrile of HPLC grade were purchased from Fisher Scientific (U.K.). Analytical grade reagents of acetic acid and boric acid were purchased from EL-Nasr,Egypt. Phosphoric acid 85% and sodium hydroxide from Sigma Aldrich (Steinheim, Germany).

### Preparation of standard solutions

An accurately weighed amount of 25 mg of each amino acid was transferred to a 25-ml volumetric flask. The amino acids were dissolved in approximately 15 mL of distilled water for Trp and 0.1 M HCl for Tyr using sonication. Complete to the mark with the corresponding solvent to prepare a stock solution of 1 mg/mL for both amino acids, was stored in the refrigerator at 4   °C. A serial dilution containing mixture of both amino acids was prepared using methanol as a diluent to obtain working concentrations of 5.0—40.0 µg.mL^−1^ and 10.0—100.0 µg.mL^−1^ for Trp and Tyr, respectively. Working solutions were prepared freshly (day by day) from the stock solutions.

### Control and diabetic rats

A total of 12 male rats from three months ago, weighing about 200–300 g, were used in this study. They were supplied from the Animal House, Faculty of Medicine, Assiut University. The research protocol was approved by the Institutional Ethics Committee at the Faculty of Medicine, Assiut University (***IRB approval no. 04-2023-200151***), which ensures compliance with the guidelines for the appropriate care and use of laboratory animals. They are divided into two groups: the experimental cases (n = 7) and the control cases (n = 5). Rats were maintained under standard conditions in a cage system for a period of 5 days prior to the beginning of the experiment. Before the administration of Streptozotocin (STZ), the rats are usually fasted overnight to ensure consistent blood glucose levels at the beginning of the experiment. Streptozotocin (STZ) (Sigma-Aldrich) was taken at a dose of 60 mg/Kg body weight, according to previous reports [16]. The solution of the calculated dose for each rat was prepared in citrate buffer pH 4.0 (freshly prepared, 100 mmol L^−1^ citric acid mixed with 100 mmol L^−1^ sodium citrate).

In the group of experimental cases, diabetes was induced by a single intraperitoneal injection of STZ at the calculated dose, where rats were deprived of food 6 h prior to administration of STZ. Blood samples were collected prior to STZ administration (on day 0) as well as on three consecutive days. After the collection of blood samples, the serum was obtained by centrifugation at 5000 rpm for 20 min. 300 μl of serum sample were transferred to 2.0 mL Eppendorf tube. 150 μl of 5% perchloric acid was also added to precipitate the serum protein, then vortexed for 1 min, followed by centrifugation at 14000 rpm for 30 min at 4   °C. The supernatant was collected into another clean Eppendorf tube, and 50 μl of 0.2 N NaOH was added to reduce the acidity. Finally, 10 μl of standard Trp (1.0 mg. ml^−1^) and 30 μl of standard Tyr (1.0 mg. mL^−1^) were added to the solution mixture, followed by dilution to a final volume of 0.5 ml using distilled water.

### Chromatographic conditions

The plates were pre-washed with methanol and left to dry at room temperature. In the TLC tank, 90 mL of the freshly prepared mobile phase were added. It consists of acetonitrile, ethyl acetate, and Britton-Ribonson buffer with a pH of 9 (50:20:20 v/v/v). Then it was covered with aluminum foil, followed by the lid, and then left for saturation at room temperature for 25 min. Five microliters of the working standards or samples were applied to the plate using a Linomat-5 automatic sample applicator with bands of 4 mm in width and 10 mm away from each edge and from the bottom. After that, the plate was allowed to develop in an ascending mode until it reached the solvent with a development distance of 65 mm. After development, the plate was left to dry in the air, then it was scanned using Camag TLC scanner 4 with WinCATS version 1.4.10 software in the absorbance and fluorescence modes at the dual wavelengths of 280 and 225 nm for both absorption and excitation, and filter k320 was selected in the fluorescence mode.

### Multi-criteria decision tools:

For the analysis of optimization data and model validation, both Microsoft Office 2016 Excel and Minitab version 17.1.0 program packages were utilized. The experimental data was subjected to multiple regression analysis to estimate the selectivity of the responses under investigation as a function of the examined variables. The optimal design was determined with the assistance of selected criteria.

### Method validation

The proposed method was validated according to ICH guidelines [17] with respect to selectivity, linearity, range, precision (intraday and inter-day), accuracy, limits of detection and quantitation (LOQ), and robustness.

#### Selectivity

The separation factor (α) was used as a measure for the selectivity of separation in a chromatographic system, and it was calculated from the following equation: [18]

$$\alpha \, = \, [({1}/{\text{R}}_{{{\text{f1}}}} ) \, - { 1}\left] / \right[({1}/{\text{R}}_{{{\text{f2}}}} ) \, - { 1}]$$

(Where R_f1_ and R_f2_ are the retardation factors for more silica-retained analyte and less silica-retained analyte, respectively). Its value should be more than 1.0 to verify a good separation.

Also, the determination of the resolution factor (R_S_) is important to estimate the accuracy of the method for quantitative analysis, and it could be calculated by the following equation: [19]

$${\text{R}}_{{\text{S}}} = \, \left( {{\text{z}}_{{1}} - {\text{z}}_{{2}} } \right)/\left( {0.{5}\left( {{\text{w}}_{{1}} + {\text{w}}_{{2}} } \right)} \right)$$

(Where z_1_ and z_2_ are the separation distances of the adjacent peaks 1 and 2, w_1_ and w_2_ are the widths of two adjacent peaks 1 and 2**)**. Its value should exceed 1.0 to implicate a good resolution.

In addition, the number of theoretical plates (N) and the height equivalent to the theoretical plate (H or HETP) are parameters that also determine the separation efficacy of the chromatographic system utilized and the extent of broadening of the analyte spot. They are calculated by the following equations: [19]

$${\text{N }} = \, \left( {{16}.\iota .{\text{z}}} \right)/{\text{w}}^{{2}} ,{\text{N }} = \, \left( {\iota /{\text{H}}} \right)$$

(Where ɭ and z are the migration distance of the mobile phase and the solute, respectively, and w is the width of the chromatographic spot (we used the width of the chromatographic peak).

The small H value and maximum N value indicate the high efficiency of the used chromatographic system regarding: smaller particle size of the stationary phase, the low flow rate of the mobile phase, the less viscous mobile phase, and small solute molecules [19].

#### Linearity and range

To verify the linearity and range of the proposed method, a group of six calibration mixtures were analyzed in concentration ranges of 25–200 ng band^−1^ for Trp and 50–500 ng band^−1^ for Tyr. A linear least squares regression analysis was used to process the peak area and amino acid concentration values in order to construct the calibration curve. The regression parameters were determined, and the analysis of serum samples was validated based on the results obtained from each regression equation for the respective compound.

#### Limits of detection (LOD) and Limit of Quantitation (LOQ)

The LOD is the smallest concentration of the analytes that can be detected without appropriate accuracy. While the LOQ is the smallest concentration of the analyte that can be determined with a certain precision and accuracy. Both parameters are essential and need to be calculated. The LOD and LOQ values were calculated based on the calibration curve results, respectively, as follow: ${\text{LOD }} = \, \left( {\left( {{3}.{3} \times {\text{S}}_{{\text{b}}} } \right)/{\text{a}}} \right)$ and ${\text{LOQ }} = \, \left( {\left( {{1}0 \times {\text{S}}_{{\text{b}}} } \right)/{\text{a}}} \right)$.

Where S_b_ is the standard deviation of the intercept and a is the slope of the calibration curve.

#### Precision and accuracy

The precision of the proposed method was evaluated for both intra-day (repeatability) and inter-day (intermediate precision) variations. The precision study involved three concentration levels (low, medium, and high) of the compounds under investigation. In order to assess intra-day precision, six replicates of each concentration were prepared and developed under the same conditions as described earlier in Sec. "Chromatographic conditions".

For inter-day precision, six replicates of each concentration were chosen from the three levels chosen by performing the analysis over three consecutive days. Precision was expressed as the relative standard deviation (RSD%), while accuracy was determined as the percentage recovery mean.

#### Robustness

To evaluate the robustness of the proposed method, the impact of minor variations on the peak area of the band was studied. This was achieved by analyzing the effect of experimental condition variations on the analysis, such as changes in mobile phase compositions, (± 0.1 mL), pH of the mobile phase (± 0.1), saturation time (± 2 min), migration distance of the mobile phase (± 2 mm) and the detection wavelength (± 2 nm).

## Results and discussion

### Spectral analysis

**Fig. 1:**
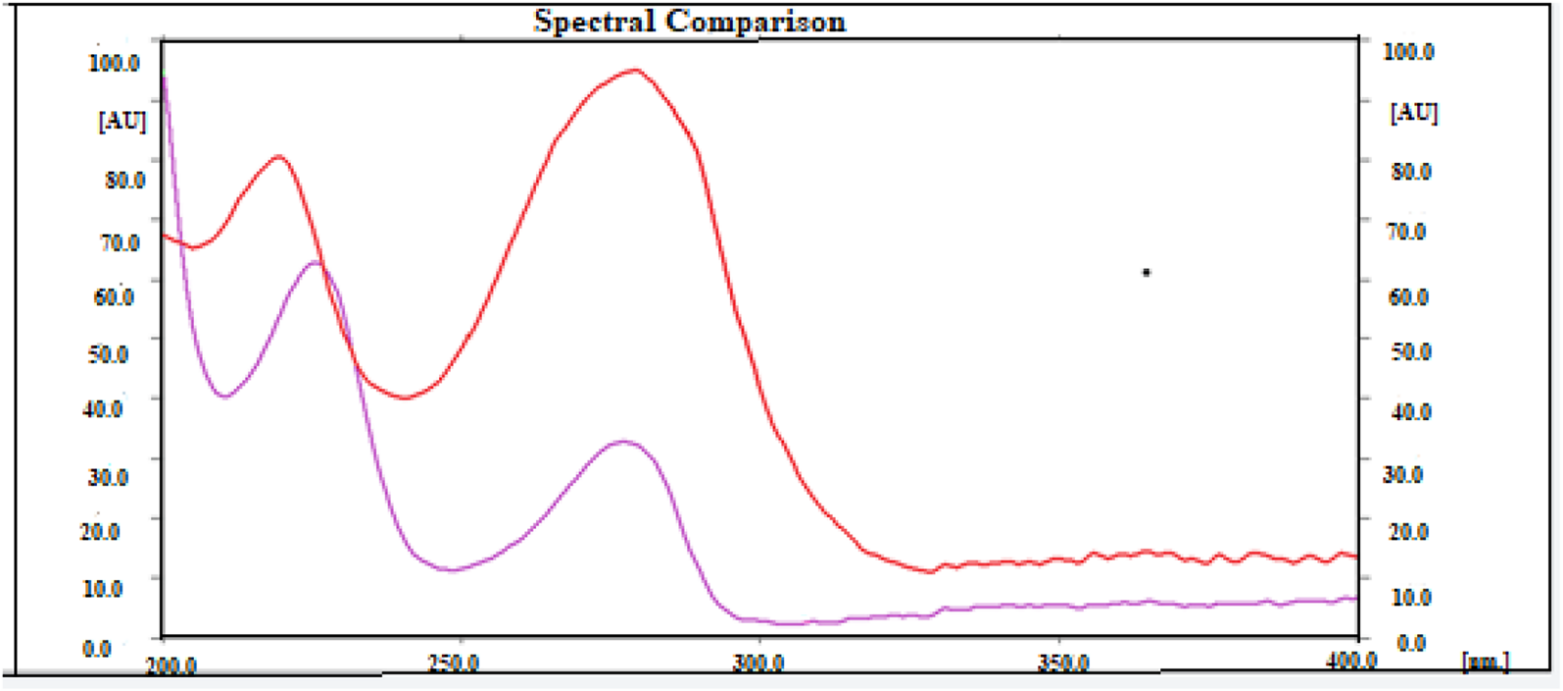
Absorption spectrum of 100 ng band^−1^Trp (red curve) and 300 ng band^−1^Tyr (violet curve) measured on HPTLC plate

The absorption spectra for both amino acids were scanned in the range 200–400 nm after TLC plate development. The obtained data showed that both amino acids have two absorption maxima. Trp has a high absorption peak at 280 nm and a second small peak at 220 nm, while Tyr has a high absorption peak at 225 nm and a second small peak at 280 nm, as shown in Fig. 1.

Therefore, the peak at 280 nm was selected for Trp and 225 nm for Tyr for scanning at both absorption and fluorescence modes.

### Optimization of the chromatographic conditions

#### Mobile phase compositions

The separation of Trp and Tyr is based on the polarity of the side chain, depending on the nature of the analytes as amino acids. The indole moiety of Trp is less polar than the phenolic moiety of Tyr. So Trp is less bound to silica than Tyr, and the ionization of the hydroxyl group of Tyr makes it more bonded to silica.

**Fig. 2:**
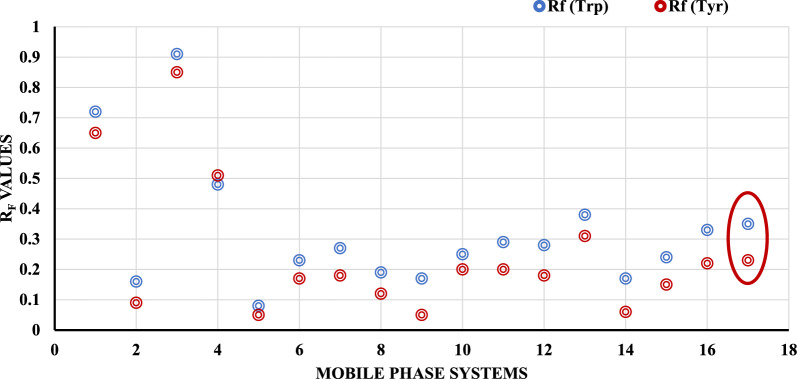
**A** comparison chart showing the differences between the values of Rf of both Trp and Tyr in the different tested solvent systems (1–17) stated in Table 1

Several trials for the developing system were performed to separate the two amino acids, as shown in Table 1 and Fig. 2.

**Table 1:** Mobile phases tested for the separation of Trp and Tyr mixtures

System	Components	Ratio (by volume)	Rf (Trp)	Rf(Tyr)
1	n-propanol: ethylene glycol: ethyl acetate	5.0:3.0:2.0	0.72	0.65
2	Chloroform: methanol: glacial acetic acid	8.5:1.4:0.1	0.16	0.09
3	Acetonitrile: water	5.0:5.0	0.91	0.85
4	n-hexane: n-propanol: glacial acetic acid	2.0:7.0:1.0	0.48	0.51
5	Acetonitrile: ethyl acetate	7.0:2.0	0.08	0.05
6	Acetonitrile: ethyl acetate	6.0:2.0	0.23	0.17
7	Acetonitrile: ethyl acetate	5.0:2.0	0.27	0.18
8	Acetonitrile: ethyl acetate	4.0:2.0	0.19	0.12
9	Acetonitrile: ethyl acetate:25%ammonia	6.0:2.0:0.3	0.17	0.05
10	Acetonitrile: ethyl acetate: BR buffer pH 7.0	5.0:2.0:1.0	0.25	0.2
11	Acetonitrile: ethyl acetate: BR buffer pH 8.0	5.0:2.0:1.0	0.29	0.2
12	Acetonitrile: ethyl acetate: BR buffer pH 9.0	4.0:2.0:1.0	0.28	0.18
13	Acetonitrile: ethyl acetate: BR buffer pH 9.0	6.0:2.0:1.0	0.38	0.31
14	Acetonitrile: ethyl acetate: BR buffer pH 9.0	5.0:3.0:1.0	0.17	0.06
15	Acetonitrile: ethyl acetate: BR buffer pH 9.0	5.0:2.0:0.5	0.24	0.15
16	Acetonitrile: ethyl acetate: BR buffer pH 9.0	5.0:2.0:1.0	0.33	0.22
17	Acetonitrile: ethyl acetate: BR buffer pH 9.0	5.0:2.0:2.0	0.35	0.23

As shown in the table, the mobile phases containing a high percentage of nonpolar solvents, such as chloroform did not improve the separation and their elution strengths for both amino acids were very low. Also, the use of a mobile phase containing n-hexane with n-propanol and glacial acetic acid improved the elution strength as the nonpolar solvent percentage was reduced. However, the separation efficiency has not improved.

On the other hand, when using high-polar mobile phases such as acetonitrile and water with an equal percentage or high percentage of methanol and a mixture of tetrahydrofuran and glacial acetic acid, the separation efficiency was not improved, and the elution strength was very high. Therefore, with the use of polar aprotic solvents as acetonitrile and moderate polar solvents as ethyl acetate in ratio of 6:2 the separation efficiency slightly increased, but the elution strength was still low. When glacial acetic acid and ammonia were added to the previous system (acetonitrile: ethyl acetate), no improvement was also observed in the separation efficiency with glacial acetic acid or the elution strength for ammonia. Therefore, we tried using BR buffer as a pH adjuster. As a result, different pH values were tried. It was observed that pH 9.0 or higher was more efficient in enhancing the separation efficiency with reasonable elution strength.

In addition to the adjustment of both acetonitrile and ethyl acetate volumes. Therefore, we used the experimental design approach to undergo the screening and optimization stages.

We selected each of acetonitrile volume, ethyl acetate volume, buffer volume and the pH of the selected solvent system as the most effective factors in the resolution process. The selection of factors is based on the preliminary experiments alone. The design matrix used for our study depends upon the selected factors along with experimentally determined and calculated responses as given in Table 2. The ranges of the independent factors used were acetonitrile volume (4.0–7.0), ethyl acetate volume (2.0–3.0), buffer pH (6.4–11.0) and buffer volume (0.0–2.0) which represented by X_1_, X_2_, X_3_ and X_4_ respectively.

**Table 2:** Tested mobile phases and design matrix used for chromatographic method optimization with their experimentally obtained responses

Exp. number	Factor levels				Responses				
	ACN V	pH	Buffer V	E Ac V	Rf		N		Rs
					Rf (Trp)	Rf (Tyr)	N (Trp)	N (Tyr)	
	X1	X2	X3	X4	Y1	Y2	Y3	Y4	Y5
1	7	6.4	0	2	0.08	0.05	261.2	148.4	0.42
2	6	6.4	0	2	0.23	0.17	751.0	504.5	0.84
3	5	6.4	0	2	0.27	0.18	881.6	534.2	1.25
4	4	6.4	0	2	0.19	0.12	620.4	356.1	0.98
5	6	11	0.3	2	0.17	0.05	555.1	148.4	1.67
6	5	7	1	2	0.25	0.2	816.3	593.5	0.625
7	5	8	1	2	0.29	0.2	946.9	593.5	1.25
8	4	9	1	2	0.28	0.18	914.3	534.2	1.39
9	6	9	1	2	0.38	0.31	1240.0	873.3	0.976
10	5	9	1	3	0.17	0.06	555.2	178.0	1.53
11	5	9	0.5	2	0.24	0.15	783.7	445.1	1.25
12	5	9	1	2	0.33	0.22	1077.6	652.9	1.5
13	5	9	2	2	0.35	0.23	1142.9	685.5	1.67

The selected responses (dependent variables) were Rf of Trp, Rf of Tyr, N Trp, N Tyr and Rs which represented by Y_1_-Y_5_ respectively.

**Fig. 3:**
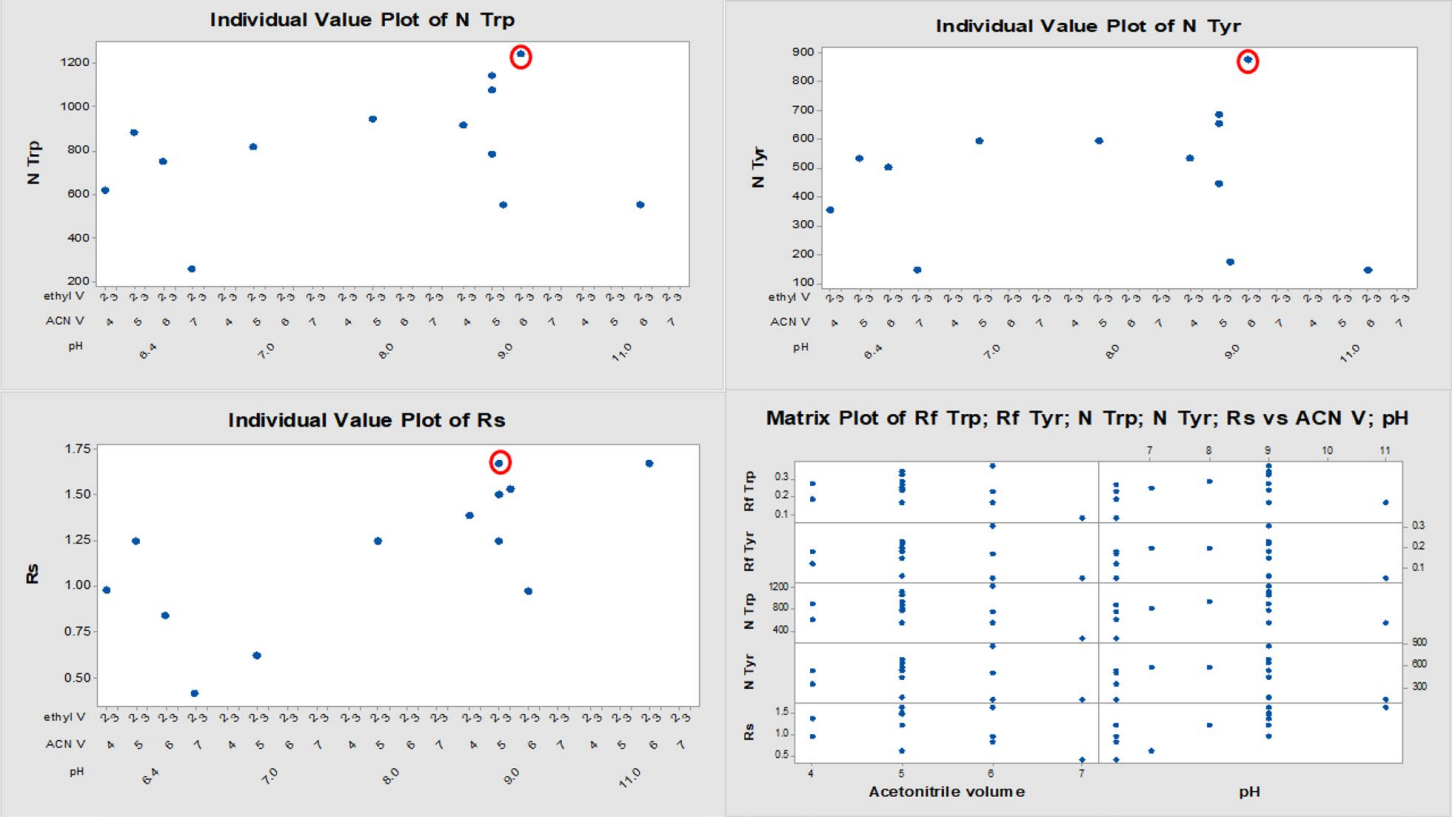
Draftman's (matrix) plot and individual plots for the responses Y1-Y5 versus the most effective chromatographic variables (number of theoretical plates (N), resolution (Rs) and pH)

**Fig. 4:**
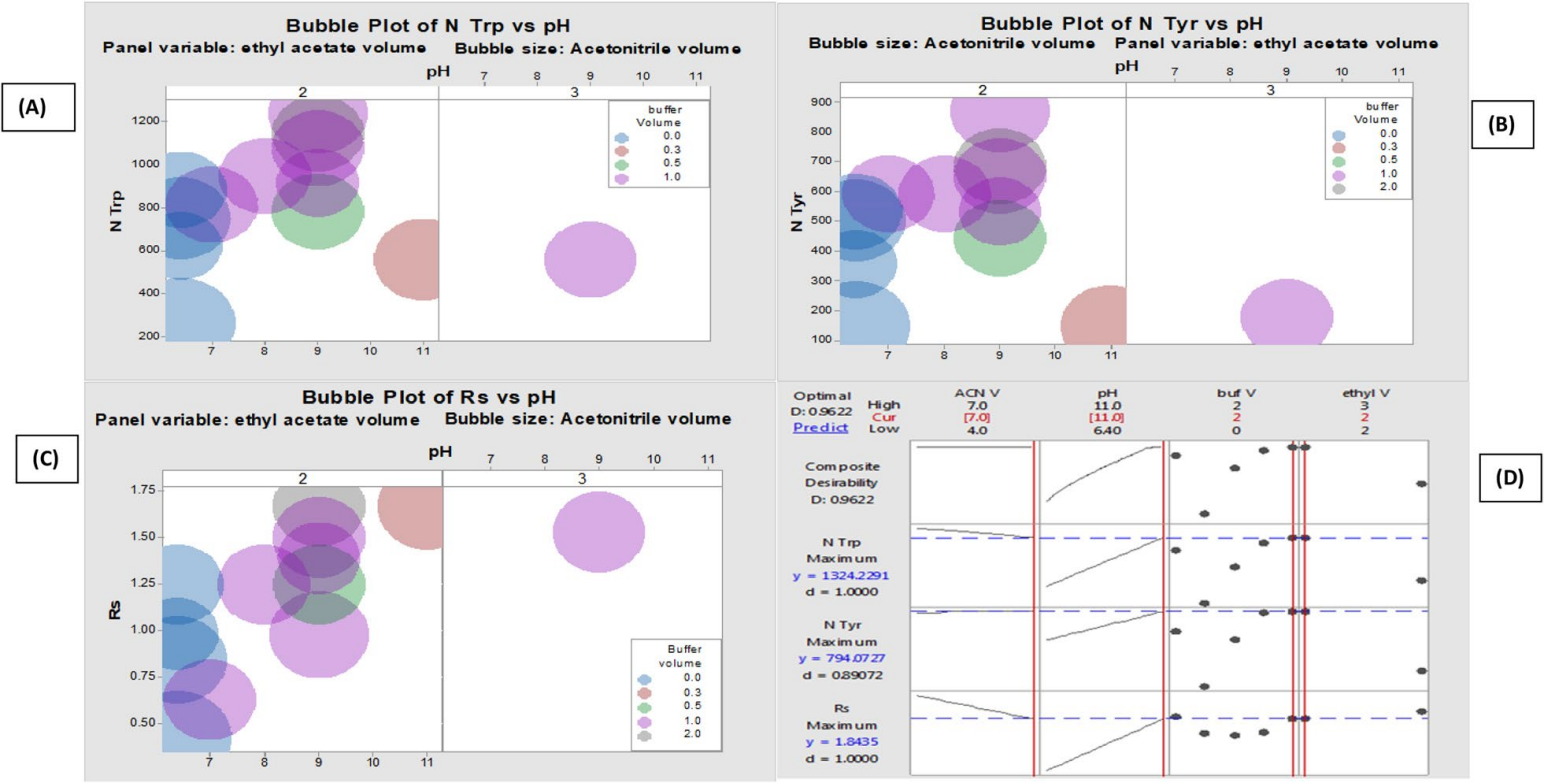
The composite desirability plot (**A**) and Bubble plots (**B**–**D**) for the chromatographic responses (N Trp, N Tyr and Rs) versus the most effective variables

As seen by the matrix plot, and the individual response plots **(**Fig. 3**)**, there are indicative relationships between all pairs of studied variables (responses versus independent variables). The red-marked readings indicate the most optimal ones at the given experimental conditions. The design space of the selected model for each response was supported using the bubble plots for the chromatographic responses versus the most effective variables (Fig. 4 A, B, C). The overall (composite) desirability approach is used as a very useful parameter for the good selection of the given variable ranges. As the value of D is close to 1. This means that all chromatographic responses are in the desired range. On the other hand, when D is close to zero, this indicates that one or more responses are outside the acceptable limits (Fig. 4D).

From the given data, we see that D value is 0.96 which is close to 1 and consequently the optimized experimental conditions fit the desired model.

Therefore, the optimal separation variables using all the subsequent work are acetonitrile: ethyl acetate: BR buffer pH 9.0 (5:2:2 V/V/V).

**Fig. 5:**
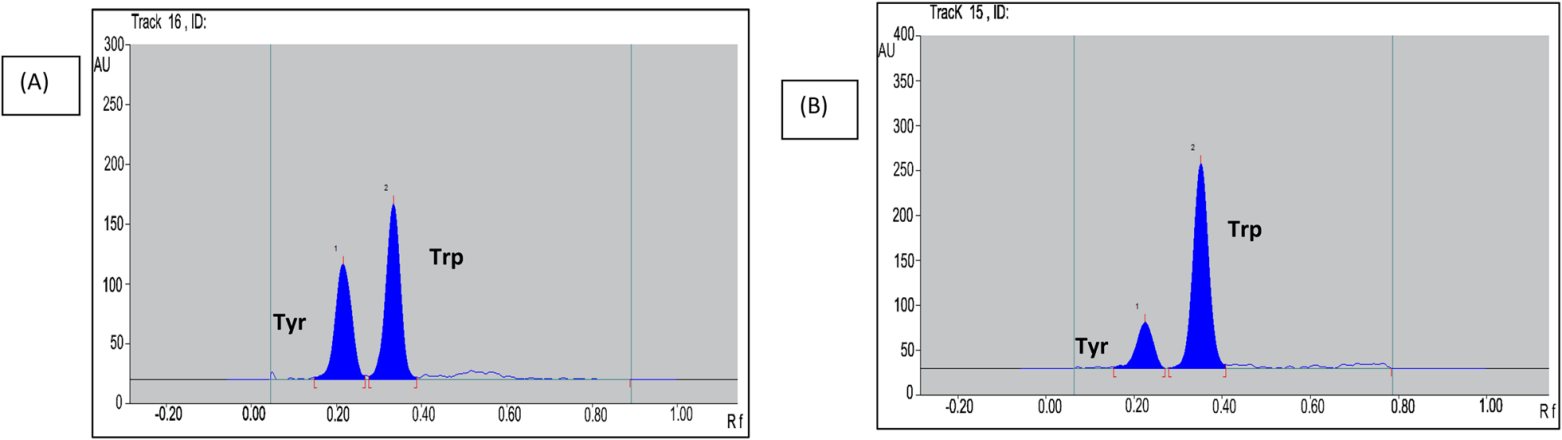
Two-dimensional TLC densitogram of mixture containing 200 ng.band^−1^ of Trp (Rf = 0.35) and 500 ng.band^−1^(Rf = 0.23) using acetonitrile: ethyl acetate: BR buffer pH 9.0 in the ratios 5:2:2, v/v/v (**A**) at 280 nm in the absorbance mode (**B**) in the flourescence mode

The separation pattern (obtained densitograms) using the selected mobile phase was shown in Fig. 5 for both absorbance and fluorescence modes, respectively.

#### Migration distance

The effect of migration distance was also optimized to achieve reasonable Rf values. The ideal distance was determined to be 65 mm from the start line, which was covered by the mobile phase in approximately 6 min.

#### Saturation time

The level of saturation of mobile phase vapors was optimized to ensure consistent saturation levels across all measurements and obtain reproducible results. Different saturation times, ranging from 15–30 min, were tested, and the optimal saturation time was determined to be approximately 25 min.

### Method validation

#### Selectivity

The selectivity of the proposed method for the separation of two adjacent peaks of Trp and Tyr is determined by separation factor α which can be calculated by the following equation: [18]

$${{\varvec{\upalpha}}} \, = \, \left[ {\left( {{\mathbf{1}}/{\mathbf{R}}_{{{\mathbf{f1}}}} } \right) \, - \, {\mathbf{1}}} \right]/\left[ {\left( {{\mathbf{1}}/{\mathbf{R}}_{{{\mathbf{f2}}}} } \right) \, - \, {\mathbf{1}}} \right]$$

where R_f1_ and R_f2_ are the retardation factors for Tyr and Trp, respectively. Its value should be higher than 1 for good separation. When it was calculated, it was found to be 1.8 which verified a good separation.

It is important also to determine the value of the resolution factor (R_S_) to ensure the good selectivity and accuracy of the proposed method for quantitative analysis of Trp and Tyr.

The resolution value (R_S_) can be calculated by the following equation: [19]

$${\mathbf{R}}_{{\mathbf{S}}} = \, \left( {{\mathbf{z}}_{{\mathbf{1}}} - {\mathbf{z}}_{{\mathbf{2}}} } \right)/\left( {{\mathbf{0}}.{\mathbf{5}}\left( {{\mathbf{w}}_{{\mathbf{1}}} + {\mathbf{w}}_{{\mathbf{2}}} } \right)} \right)$$

where z_1_ and z_2_ are the migration distances of Trp and Tyr, respectively, and w_1_ and w_2_ are the widths of the two adjacent peaks of Trp and Tyr, respectively.

The resolution factor (R_S_) determines the possibility of resolution between the two adjacent peaks of Trp and Tyr, and it should be more than 1. On the calculation of the resolution factor, its value was 1.67 which imparts an acceptable resolution of our chromatographic system.

Moreover, the broadening of the chromatographic spot and the separating efficiency of the chromatographic plates can be estimated in terms of the number of theoretical plates N of the chromatographic system using the following equation: [19]

$${\mathbf{N}} \, = \, \left( {{\mathbf{16}}.\iota .{\mathbf{z}}} \right)/{\mathbf{w}}^{{\mathbf{2}}}$$

where ɭ and z are the migration distances of the mobile phase and the solute (Trp and Tyr), respectively, and w is the width of the chromatographic spot (we used the width of the chromatographic peak). The calculated values for N were 1142.85 plates for Trp and 682.5 plates for Tyr.

Additionally, the separating efficiency of the used chromatographic system can be expressed by calculating H or HETP (height equivalent of a theoretical plate) from the following equation: [19]

$${\mathbf{N}} \, = \, \left( {\iota /{\mathbf{H}}} \right)$$

The calculated values for H were 0.003 cm plate^−1^ for Trp and 0.005 cm plate^−1^ for Tyr.

The obtained results for N and H indicate the high separation efficiency of our used chromatographic system.

#### Linearity and range

**Fig. 6:**
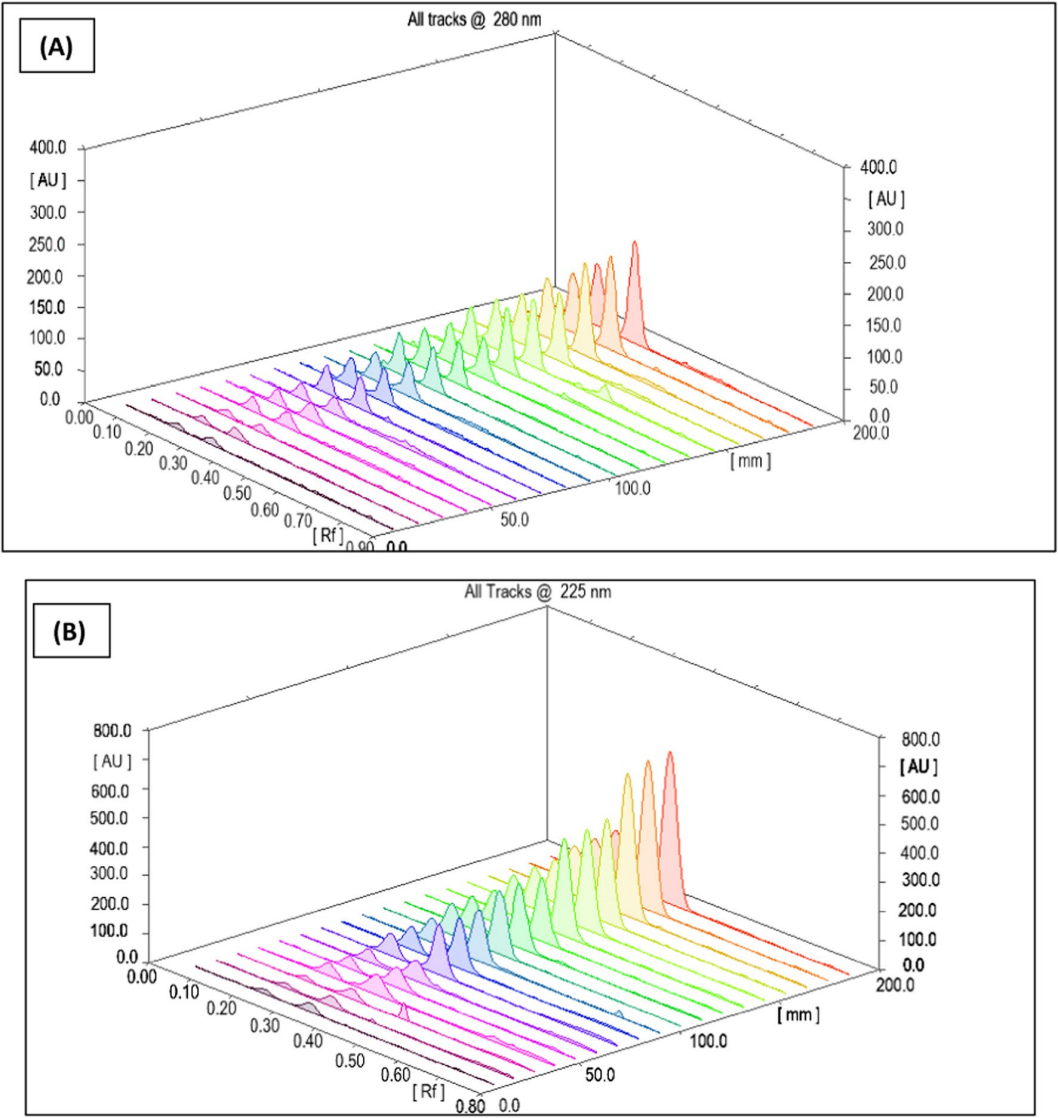

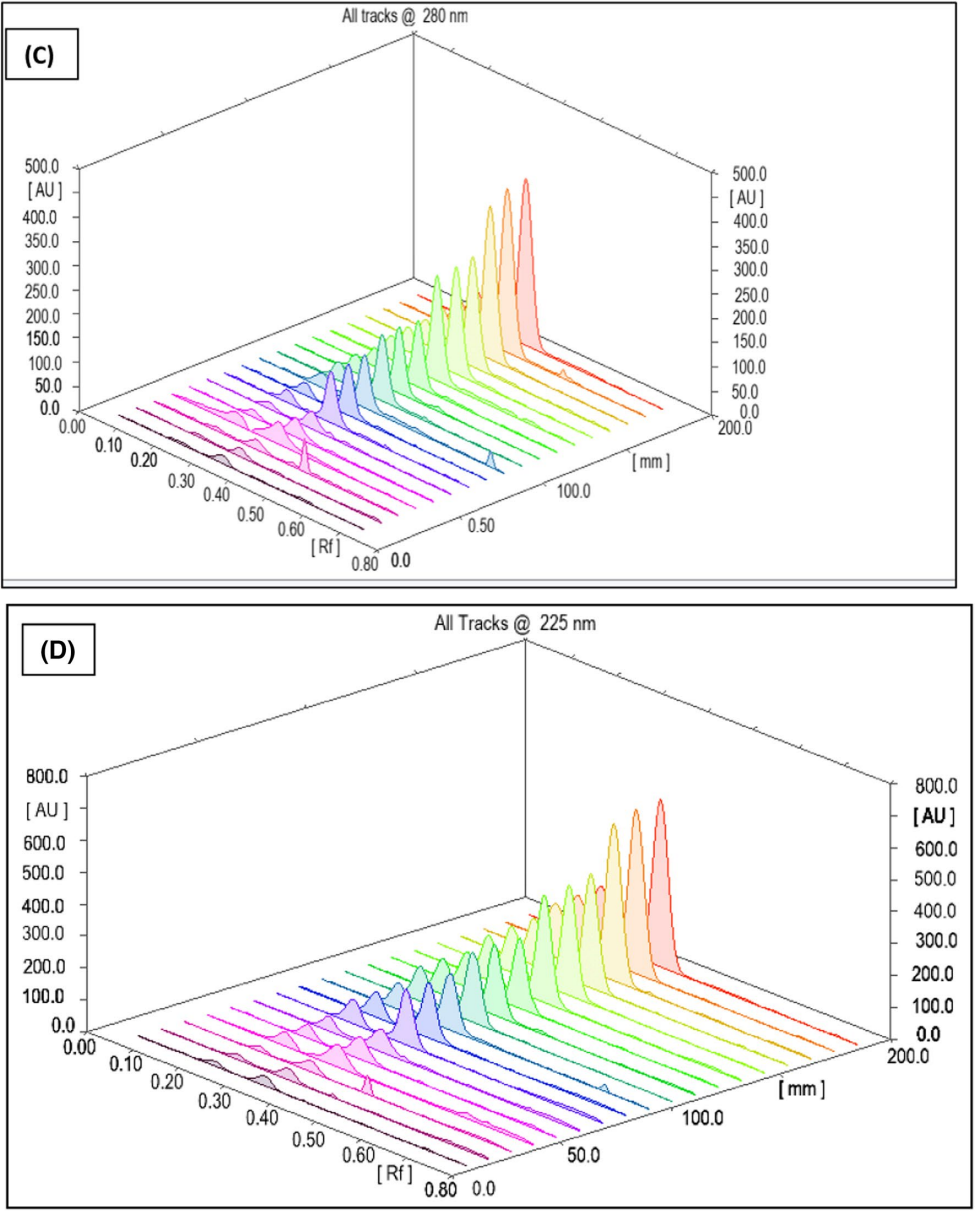
Three-dimensional densitogram for calibration of the integrated analyte peaks against Rf values for six calibration levels of Trp and of Tyr at (**A**) 280 nm for UV detection (**B**) 225 nm for UV detection (**C**) 280 nm with filter K320 for fluorescence detection (**D**) 225 nm with filter K320 for fluorescence mode

To construct the calibration curves for the two analytes under investigation, the integrated peak area was plotted against the analyte concentration. Six concentration levels were examined for each analyte (Trp and Tyr), with three replicates from each level. The resulting calibration curves can be seen in Fig. 6. The investigated linearity parameters are listed in Table 3**.**

**Table 3:** Linearity parameters for the absorbance and fluorescence mode detection

	Absorbance mode		Fluorescence mode	
Parameter	Trp	Tyr	Trp	Tyr
Excitation wavelength(nm)	280	225	280 with filter K320	225 with filter K320
R_f_	0.35 ± 0.05	0.23 ± 0.05	0.35 ± 0.05	0.23 ± 0.05
Experimental estimated range (ng. band^−1^)	25–200	50–500	25–200	50–500
Intercept (a) ± SD^*^	−74.05 ± 3.67	89.13 ± 11.58	−510.66 ± 22.2	24.97 ± 11.66
Slope (b) ± SD*	12.06 ± 0.44	6.49 ± 0.19	28.36 ± 0.55	2.77 ± 0.05
r^**^	0.9990	0.9987	0.9994	0.9998
r^2***^	0.9982	0.9976	0.9987	0.9996
LOD (ng/band)	1.004	5.97	2.59	13.88
LOQ (ng/band)	3.04	18.08	7.86	42.05

^*^SD when n = 3

^**^r is the correlation coefficient

^***^r^2^ is the determination coefficient

#### Precision and accuracy

The precision of the proposed method was evaluated at three concentration levels (50,100,150 ng band^−1^ for Trp) and (100,300,400 ng band^−1^ for Tyr) covering the low, medium and high concentration levels.

Six replicates of these concentrations for both analytes were determined on the same day and on three consecutive days for the intra-day and inter-day precision of the proposed method. The precision was evaluated in term of %RSD. For all concentration levels the %RSD did not exceed 4.68 which proved the good precision of the proposed method. The accuracy for both analytes (Trp and Tyr) was determined as the percentage recoveries mean as indicated in Table 4.

**Table 4:** Summary for the precision and accuracy of the proposed method

Amino acids	Conc. (ngband^−1^)	Absorption mode				Fluorescence mode			
		Intra-day precision (n = 6)		Inter-day precision (n = 18)		Intra-day precision (n = 6)		Inter-day precision (n = 18)	
		%Recovery ± SD*	RSD%	%Recovery ± SD*	RSD%	%Recovery ± SD*	RSD%	%Recovery ± SD*	RSD%
Trp	50	98.92 ± 1.43	3.22	100.48 ± 0.95	4.78	98.26 ± 0.88	3.17	100.79 ± 0.35	4.68
	100	98.09 ± 0.02	2.77	100.01 ± 1.15	4.29	99.60 ± 0.17	0.53	99.68 ± 0.83	4.67
	150	98.13 ± 0.55	1.71	99.43 ± 1.65	3.01	99.29 ± 0.32	1.08	100.79 ± 0.89	4.77
Tyr	100	98.07 ± 0.78	4.12	100.64 ± 0.58	4.11	99.27 ± 1.69	1.81	98.75 ± 0.85	3.39
	300	99.30 ± 0.78	1.41	99.99 ± 0.26	3.35	98.86 ± 0.43	1.17	99.25 ± 0.93	2.34
	400	99.43 ± 0.29	1.78	100.01 ± 0.49	3.74	99.16 ± 0.93	3.80	98.72 ± 1.36	4.00

The values of percentage recoveries were in the range of (98.09–100.79%) for Trp and (98.07–100.64%) for Tyr indicating the high accuracy of the developed method

^*^SD when n = 3

#### Robustness

Different effective parameters were studied and changed slightly to measure the ability of the proposed method to remain unaffected by these small variations that indicate the method reliability during normal conditions.

The robustness of our method was studied using 150 ng band^−1^ for Trp and 300 ng band^−1^ for Try. The effect of variation in detection wavelength (± 2 nm), mobile phase composition (± 0.1 mL), pH of the mobile phase (± 0.1), saturation time (± 2 min.) and the migration distance (± 2 mm) were studied. The results were expressed as % Recovery ± SD as listed in Table 5 for both absorbance and fluorescence modes.

**Table 5:** Robustness of the proposed method at concentrations of 150 ngband^−1^ for Trp and 300 ngband^−1^ for Try for absorbance and fluorescence mode detection

Investigated parameters	Absorbance mode				Fluorescence mode			
	Trp		Tyr		Trp		Tyr	
No variation*	100.98 ± 1.26		99.09 ± 0.29		98.70 ± 0.30		100.31 ± 0.67	
Detection wavelength	278 nm	99.99 ± 0.3	223 nm	103.35 ± 1.10	278 nm	98.91 ± 0.52	223 nm	98.78 ± 0.14
	282 nm	100.34 ± 0.7	227 nm	99.45 ± 1.06	282 nm	100.35 ± 2.02	227 nm	102.03 ± 0.07
Mobile phase compositions (Acetonitrile: ethyl acetate: BR buffer pH 9.0)	4.9: 2.1:2	101.88 ± 3.22	100.43 ± 1.51		97.72 ± 1.19		99.57 ± 0.74	
	5.1:1.9:2	98.07 ± 1.12	95.78 ± 2.04		99.29 ± 0.61		97.48 ± 0.11	
pH of the mobile phase	8.9	100.04 ± 2.18	100.87 ± 2.89		98.82 ± 0.96		97.12 ± 1.77	
	9.1	105.07 ± 1.96	99.58 ± 0.13		100.13 ± 2.49		99.49 ± 0.74	
Migration distance	63 mm	99.93 ± 0.11	100.16 ± 0.31		101.57 ± 1.44		100.91 ± 1.13	
	67 mm	98.38 ± 1.99	100.32 ± 1.21		100.59 ± 2.67		98.80 ± 1.14	
Saturation time	23 min	99.96 ± 1.58	99.56 ± 0.05		99.74 ± 1.17		100.83 ± 0.91	
	27 min	100.29 ± 2.26	101.06 ± 0.92		99.55 ± 1.18		97.78 ± 0.76	

^*^No variation means using all the optimum conditions

### Application to rat serum samples

As mentioned before, Trp degradation by the kynurenine pathway was suggested to increase in T2D due to the increase in the activity of indoleamine-2,3-dioxygenase (IDO) enzyme [7, 9], and also the stress condition that affects the diabetic patient increase Trp degradation [10]. Therefore, Trp serum level is significantly reduced in the case of T2D diabetes making it a potential biomarker for the evaluation of the disease condition. On the other hand, Tyr degradation was suggested to be suppressed in T2D diabetes. The suppression of its degradation is probably due to a reduction in the tyrosine transaminase enzyme, which leads to an increase in insulin resistance [12]. Another suggested behaviour for Tyr in T2D cases was the reduction in tyrosine hydroxylase enzyme activity which led to a reduction in dopaminergic agents and low anti-incretin effect [13]. Therefore, both the increase in Tyr serum levels and the decrease in Trp in T2D make them a dual potential biomarker for the evaluation of the disease.

From several previous studies, it was found that protein precipitation has been known as an efficient method in chromatographic determination of amino acids in plasma or serum [20–23]. Different precipitating agents were studied as methanol, acetonitrile and perchloric acid. Perchloric acid gave the best densitogram for both amino acids. These results agree with the previous results reported by Sedgwich and his co-workers who studied in detail the effect of different protein precipitating agents on the recovery of different amino acids including our studied amino acids Trp and Tyr [24].

Perchloric acid in different concentrations was studied. The concentrations below 5% could not be able to completely precipitate the serum protein and higher than 5% was destructive to silica. Different ratios of serum to 5% perchloric acid were tested (2:1, 1:1, 1:2). It was found that the best densitograms with good peak resolution and good recovery was with the ratio of 2:1 as increasing the ratio of perchloric acid was destructive to the stationary phase and this affected the recovery of both amino acids and affect the peak sharpness. To improve the recovery of both amino acids, the acidity of the supernatant was reduced by the addition of a small volume of 0.2N NaOH. The best and more reproducible results were attained by the addition of 50 µL of NaOH.

**Fig. 7:**
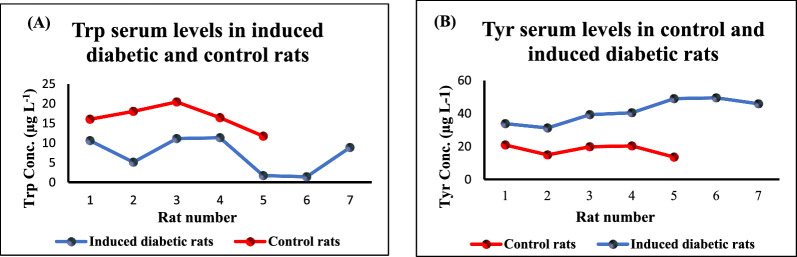
**A** Line graph for serum Trp levels in control and induced diabetic rats. **B** Line graph for serum Tyr level in control and induced diabetic rats

Control and diabetic serum rat samples were treated with the previously mentioned procedure for the determination of both amino acids as in section "Control and diabetic rats"**.** As shown in Fig.7, there was a significant decrease in Trp levels in induced diabetic rats than control rats while there was a marked increase in Tyr levels than control ones. The statistical data analysis showed that there was a significant difference (p-value<0.05) between the serum levels of Trp and Tyr in diabetic rats when compared to control rats (Table 6)

**Table 6:** the statistical comparison for control (n = 5) and diabetic rats (n = 7) serum levels of Trp and Tyr

Statistical values	Trp	Tyr
p-value*	0.006	0.0002
t-test	t = 4.28, t_critical_ = 1.81	t = 7.52, t_critical_ = 1.83
F-test	F = 0.53, F_critical_ = 0.16	F = 0.22, F_critical_ = 0.16

### Comparison to other reported methods

The performance of the reported methos was compared with the previously reported voltammetric, fluorimetric and HPLC methods as indicated in Table 7 and 8. In Table 7 the developed method was compared with some voltammetric and fluorimetric methods with different sensors [25–29]. The reported method indicated simple and green procedures with high sensitivity in biological fluids. The previously reported HPLC methods for simultaneous determination of Trp and Tyr in different matrices are presented in Table 8 [30–32]. Methods (1), (2) and (3) are applying HPLC procedures for their simultaneous determination that consume more energy. In addition to that, these methods have some disadvantages such as the consumption of large amounts of solvents in comparison with the proposed HPTLC method. Also, the use of formate buffer in the mobile phase system had a high environmental impact. And the use of highly hazardous substances in conjunction with each other of sodium metabisulfate and perchloric acid in the formation of sample homogenate is not preferred [32]. Additionally, the high energy consumption with the use of mass detection is disadvantageous for this method. While the utilization of derivatizing agent such as diethyl ethoxymethylenemalonate (which is considered a hazardous substance) increases the steps of analysis and decreases the greenness performance of that reported method [31]. For our developed method, we consume smaller amounts of solvents in comparison with the other mentioned methods. Also, it possesses fewer steps in sample preparation without the need of derivatization or utilization of highly hazardous substances in large quantities.

**Table 7:** Performance comparison of different methods for the determination of L-Tyr and Trp

Techniques	Electrode or sensor	LOD (mol L^−1^)	Matrix	Ref
DPV	(GC/RGO/ILC/CNT/Fe–Zn)	5.1 × 10^−9^ for Tyr 3.8 × 10^−9^ for Trp	Human serum	[25]
DPV	SPCE/GO–COOH–Chitosan	0.05 × 10^−6^ for Tyr 0.1 × 10^−6^ for Trp	Milk	[26]
DPV	Nafion/TiO_2_-GR/GCE	2.3 × 10^−6^ for Tyr 0.7 × 10^−6^ for Trp	Human serum	[27]
Fluorimetry	MOF@CdTe SiO_2_@MIP capillary sensor	8.0 × 10^−11^ for Tyr	Human urine and blood samples	[28]
Fluorimetry	CHA-CuNCs) sensor	0.043 × 10^−6^ for Trp	Human serum samples and fetal bovine serum samples	[29]
TLC	HPTLC	0.98 × 10^−6^ for Trp and 6.6 × 10^−6^ for Tyr	Rat serum samples	This work

*DPV* differential pulse voltammetry, *SPCE* Screen printed carbon electrode, *GO* graphene oxide, *CHA* Coal humus acid, *MOF* Metal–organic frameworks

**Table 8:** Comparison of the proposed method with other reported methods for simultaneous determination of Trp and Tyr

	Extraction method	Extraction solvent	Derivatization	Detection method	Analyte LOD (μmol L^−1)^		Ref
					Trp	Tyr	
Method (1)	Protein precipitation (deproteination)	5% Perchloric acid	No derivatization	HPLC-FLD	0.0049	0.014	[30]
Method (2)	Deproteination through ultrafilteration inserts	HCL + TDPA	Needed	UHPLC	0.52	0.13	[31]
Method (3)	Protein precipitation	Acetonitril or mixture of (sod. Metabisulphate, perchloric acid and EDTA)	No derivatization	HPLC–MS	0.49 (plasma) 0.12 (urine) 0.0098 (Mice tissues)		[32]
Method (4)	Protein precipitation	5% perchloric acid	No derivatization	HPTLC-UV	0.98	6.6	**This work**

### Greenness assessment

In comparison the developed method with other reported chromatographic methods, our proposed method showed the highest greenness performance. Developing a green analytical method presents a significant challenge to analysts, as it requires not only achieving analytical figures of merit such as selectivity, specificity, and limit of detection, but also ensuring that the procedure is environmentally friendly. This challenge stems from the need to balance both environmental and analytical considerations in the method development process. The greenness of the developed method was evaluated using two different greenness tools which are the analytical greenness metric (AGREE) and green analytical procedure index (GAPI).

**Fig. 8:**
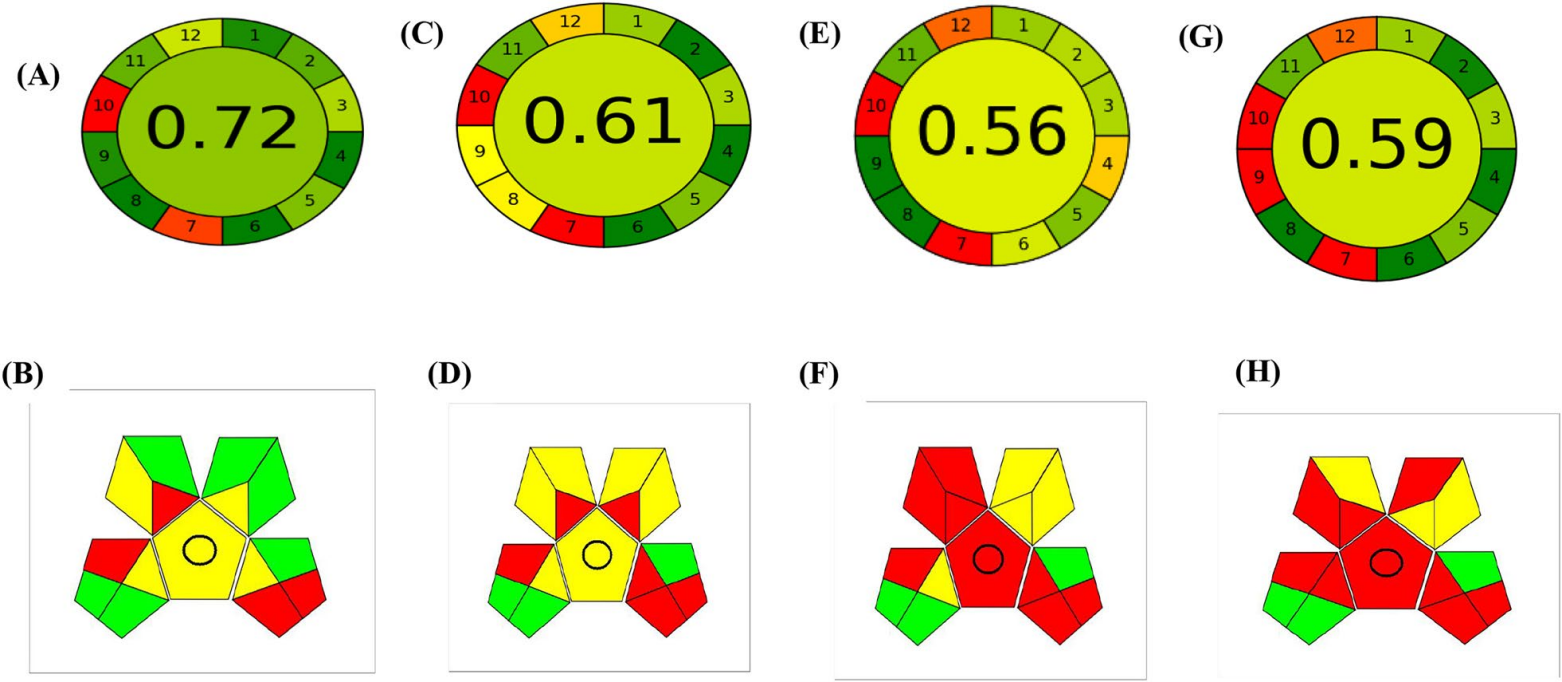
**A** and **B** AGREE result and GAPI pentagram for the proposed method, **C** and **D** for method 1, **E** and **F** for method 2, while **G** and **H** for method 3

AGREE offers a comprehensive, adaptable, and uncomplicated assessment method that produces results that are easy to interpret and informative. AGREE utilizes criteria derived from the 12 principles of GAC (green analytical chemistry), which are then converted into a unified 0-1 scale [33]. The resulting score of 0.72, as depicted in Fig. 8A, demonstrates the exceptional environmentally friendly nature of the developed approach. An eco-scale value of 0.50 or more is deemed acceptable for drug analysis, and any value below 0.50 indicates that the analytical method is unacceptable [34, 35].

When comparing the developed method with the previously reported methods [30–32] for their simultaneous determination, it could be demonstrated that the high eco-scale of our developed method as shown in Table 8 and Fig. 8**.**

GAPI is a recent addition tool to the methods used for evaluating the environmental impact of analytical procedures. It assesses the greenness of the entire analytical method, from sample collection to final determination. The tool comprises five pentagrams, each representing a different stage of the analytical process, and each stage is assigned a color code with three levels of environmental impact: green for low impact, yellow for medium impact, and red for high impact [36]. From Fig.8B, it was observed only four red regions which implicates the greenness of the developed method. Those red regions account for that special storage conditions were required for the collected serum samples as they must be frozen. The second red region was for the extraction procedure using the protein precipitation method, which is considered as macro-extraction procedure, but this method was reported as more efficient in amino acid determinations than other methods. We overcome this environmental hazard by minimizing the amount of precipitating agent to 150 μL only. The third red region was attributed to the amount of produced waste was more than 10 ml but the use of a large amount of mobile phase was required to provide proper saturation of the used development tank. The last red region accounted for no treatment for the waste but the used solvents waste as ethyl acetate and acetonitrile could be considered green solvents with low environmental hazards. The rest wastes of solvents were of very low amounts and concentrations. While on comparing the resulted pentagram with the previously reported methods, it was shown that the proposed method possesses the lowest number of red zones. Based on the eco-scale value recorded in this work, the proposed method has been found as the excellent green analytical methods for the quantitation of Trp and Tyr.

## Conclusion

An innovative simple and sensitive HPTLC densitometric method was developed for the determination of tryptophan (Trp) and tyrosine (Tyr) concentrations in the serum of control and streptozotocin-induced diabetic rats by standard addition method. The results showed that there is a significant difference in their concentrations in both studied groups. Our study provides valuable insights into the association between Trp and Tyr levels and T2D. Therefore, Trp and Tyr can be used as potential biomarkers for the evaluation of type 2 diabetes mellitus. Moreover, understanding the underlying mechanisms linking Trp and Tyr metabolism with insulin resistance could pave the way for the development of novel therapeutic strategies for T2D. The validation parameters of the developed method were found to be within acceptable limits. The calculations of separation and selectivity factors, number of theoretical plates and height equivalents of a theoretical plate verified the separation efficiency of the used chromatographic system and the accuracy for quantitative analysis. The developed method offers the property of lowering the analysis time and cost-saving as many samples can be determined simultaneously. Ultimately, the identification of reliable biomarkers and therapeutic targets related to Trp and Tyr metabolism could contribute to improved diagnosis, treatment, and management of T2D.

## Data Availability

The authors declare that the data supporting the findings of this study are available within the paper. Should any raw data files be needed in another format they are available from the corresponding author upon reasonable request.
